# Beyond antibiotics: Harnessing phage depolymerases to control multidrug-resistant Klebsiella pneumoniae

**DOI:** 10.17179/excli2025-8950

**Published:** 2025-11-14

**Authors:** Eloia Emanuelly Dias Silva, Deise Maria Rego Rodrigues Silva, Pedro Henrique Macedo Moura, Luana Ramony da Silva Lisboa, Allec Yuri Santos Martins, André Gustavo Carvalho de Oliveira, Marina dos Santos Barreto, Adriana Gibara Guimarães, Ronaldy Santana Santos, Lucas Alves da Mota Santana, Lysandro Pinto Borges

**Affiliations:** 1 Federal University of Sergipe, Northeast Biotechnology Network (RENORBIO), Av. Marcelo Déda Chagas, São Cristóvão, 49000-100 Sergipe, SE, Brazil; 2 Federal University of Sergipe, Department of Pharmacy, Av. Marcelo Déda Chagas, São Cristóvão, 49000-100 Sergipe, SE, Brazil; 3 University of São Paulo, Department of Immunology, Av. Prof. Lineu Prestes, São Paulo, 05508-900, Brazil; 4 Federal University of Sergipe, Graduate Program in Dentistry, R. Cláudio Batista, Aracaju, 49060-108 Sergipe, SE, Brazil

## ⁯

The emergence of infections caused by multidrug-resistant bacteria represents a growing challenge to global public health (WHO, 2023[12]). Among the most concerning agents is *Klebsiella pneumoniae*, a Gram-negative, facultative anaerobic bacillus possessing a thick polysaccharide capsule that enhances both its pathogenicity and its ability to form biofilms in hospital environments (Abbas et al., 2024[1]). This characteristic, coupled with its proficiency in acquiring resistance genes, has resulted in strains associated with nosocomial outbreaks of high clinical severity (Wang et al., 2020[11]).

The impact of carbapenem-resistant *K. pneumoniae* is particularly alarming. The production of the enzyme *Klebsiella pneumoniae carbapenemase* (KPC), a type of β-lactamase that inactivates last-resort antibiotics, significantly compromises available therapeutic options (Ding et al., 2023[3]). In Brazil, documented outbreaks across different states indicate that the dissemination of this microorganism is related not only to the selective pressure arising from the indiscriminate use of antibiotics but also to conditions conducive to transmission in hospitals, especially in Intensive Care Units (de Almeida et al., 2021[2]).

In this context, a concerning contradiction is observed: while the demand for novel antimicrobials is increasing, the development of new antibiotics has proven insufficient to keep pace with the evolution of bacterial resistance mechanisms (WHO, 2023[12]). Faced with this impasse, the scientific community has sought strategies based on the ecology of these organisms. Among them, phage therapy has re-emerged as a promising approach in light of current therapeutic failures (Silva et al., 2024[9]).

Bacteriophages exhibit unique attributes that make them potential allies in combating multidrug-resistant *K. pneumoniae*. Herridge (2020[4]) compiled experimental evidence demonstrating the therapeutic potential of different phages against *K. pneumoniae*. In the reviewed studies, phage Kpn5 was notable in murine models of burn wound infections with *K. pneumoniae* B5055. In another report, intranasally administered phage 1513 conferred 80 % survival in Swiss Webster mice against nasal challenge with a multidrug-resistant strain of *K. pneumoniae*, while also reducing the pulmonary lesions observed in control animals.

In a separate study cited by the author, phage SS, when administered intraperitoneally immediately after intranasal inoculation of the bacteria in BALB/c mice, resulted in complete clearance of the infection in just five days, compared to ten days in untreated animals (Herridge et al., 2020[4]). Lastly, phage KPO1K2 demonstrated efficacy in a murine model of lobar pneumonia even when administered up to three days post-bacterial inoculation, a finding that reinforces the potential of using modified delivery systems to extend the therapeutic window (Herridge et al., 2020[4]).

In addition to their specificity for bacterial hosts, many phages associated with this genus express depolymerase enzymes capable of degrading the polysaccharide capsule and penetrating biofilms, structures that often confer increased resistance to conventional antibiotics (Strathdee et al., 2023[10]). This property not only promotes direct bacterial lysis but may also enhance drug permeability, creating possibilities for combination therapies.

Depolymerases are structural components of the virion, occurring in the structural form of tail spike proteins (TSPs) (Knecht et al., 2020[6]). Accordingly, the initiation of bacterial infection by a phage begins with the recognition of specific ligands on the bacterial surface by TSPs (Knecht et al., 2020[6]). In this context, the function of these enzymes is to bind to and cleave polysaccharides on the bacterial surface, such as capsular polysaccharides (CPS), exopolysaccharides (EPS), or lipopolysaccharides (LPS) (Knecht et al., 2020[6]). Structurally, depolymerases are highly stable, protease-resistant, and highly specific enzymes, which makes them suitable for a wide range of biotechnological applications.

The study by Hua et al. (2022[5]) identified a novel depolymerase, K19-Dpo41, from a phage isolated from hospital wastewater. In experimental assays, the enzyme proved to be highly specific, degrading the capsule exclusively of K19-type carbapenem-resistant *K. pneumoniae* strains, thereby confirming its potential for capsular typing (Hua et al., 2022[5]). Recognizing the importance of these enzymes, Magill and Skvortsov (2023[7]) developed a machine learning tool capable of accurately detecting depolymerase-coding genes in complete phage genomes, thereby optimizing the search for candidate enzymes with potential applications in phage therapy.

Despite well-documented advances, the full potential of bacteriophages remains largely unexplored (Strathdee et al., 2023[10]). The characterization of new phages, involving genomic analyses, functional assays, and the assessment of morphological diversity, is an essential step toward identifying strains with greater bactericidal efficacy, broader specificity, and stability in clinical settings. Furthermore, understanding the role of depolymerases and other factors associated with phage virulence is a decisive step for their rational use in therapeutic protocols (Shaidullina and Harms, 2022[8]).

The rising incidence of multidrug-resistant *K. pneumoniae* infections demands not only continuous epidemiological surveillance but also investment in robust therapeutic alternatives. Phage therapy represents one such alternative, with inherent advantages of specificity and efficacy. However, for this potential to be effectively incorporated into clinical practice, it is imperative to intensify the search for new phages, characterize them in depth, and expand our understanding of their interactions with biofilms and bacterial resistance mechanisms. Investing in the discovery of novel bacteriophages is, therefore, more than an alternative: it is a strategic necessity in confronting antimicrobial resistance.

## Declaration

### Author contributions

EEDS, PHMM, DMRRS and LRSL conceived the study and reviewed the scientific literature. EEDS, AYSM, AGCO, RSS and MSB prepared the first draft. AGG, LAMS and LPB reviewed the paper. All authors contributed to revisions of the paper and approved the final version.

### Funding and assistance

Not applicable.

### Conflict of interest

The authors have no conflict of interest.

### Artificial Intelligence (AI) - Assisted Technology

No generative AI tools were used.
